# Genome Sequences of Four *Yersinia enterocolitica* Bioserotype 4/O:3 Isolates from Mammals

**DOI:** 10.1128/genomeA.00466-13

**Published:** 2013-07-11

**Authors:** Debora Garzetti, Jürgen Heesemann, Alexander Rakin

**Affiliations:** Max von Pettenkofer-Institute for Hygiene and Medical Microbiology, Ludwig Maximilians-University, Munich, Germany

## Abstract

We report here the complete genome sequences of four European *Yersinia enterocolitica* mammalian isolates of bioserotype 4/O:3. The genomes have an average size of 4.50 Mb, a G+C content of 47%, and between 4,231 and 4,330 coding sequences (CDSs). No relevant differences were detected by genome comparison between mammalian and human isolates.

## GENOME ANNOUNCEMENT

*Yersinia enterocolitica* is a food-borne pathogen primarily found in mammals, with humans being mostly accidental hosts. In humans, after the ingestion of contaminated food or water, *Y. enterocolitica* colonizes the intestines, most frequently causing acute gastroenteritis with fever, vomiting, and diarrhea (1).

*Y. enterocolitica* consists of a biochemically and genetically heterogeneous group of organisms, divided into 6 biotypes (the nonvirulent 1A, the highly virulent 1B, and the low-virulence 2, 3, 4, and 5 biotypes) and >70 serotypes. Human infections with *Y. enterocolitica* are documented worldwide and are mostly caused by strains belonging to the pathogenic bioserotype 4/O:3 (2). *Y. enterocolitica* has been isolated from mammals, birds, and other animal species, as well as from the environment. Swine are the primary reservoir for food-borne illness associated with *Y. enterocolitica*, mainly of that of bioserotype 4/O:3 (3).

Genomes of *Y. enterocolitica* isolates of different bioserotypes have been sequenced, allowing the identification of serotype-specific features (4–9). Among these, four European clinical isolates (4, 10) and one Philippine isolate from swine (11), all belonging to the prevailing bioserotype 4/O:3, are currently available. Whole-genome sequences of *Y. enterocolitica* 4/O:3 isolates from animal sources would provide a comprehensive knowledge of the epidemiology and transmission of this frequently encountered bioserotype.

Four *Y. enterocolitica* strains of bioserotype 4/O:3 were selected, two isolates from pig (*Y. enterocolitica* YE-P1 and YE-P4), one dog isolate (*Y. enterocolitica* YE-149), and one strain isolated from calf (*Y. enterocolitica* YE-150). For each strain, a 150-bp paired-end library was constructed and used for whole-genome sequencing by the Illumina MiSeq technology (IMGM Laboratories, Martinsried, Germany). The run produced from 2.02 to 5.41 million reads, having an average length ranging from 142.44 to 147.34 bp and an average Phred quality score of 37.

Mapping assemblies using the published complete genome sequence of *Y. enterocolitica* strain Y11 (accession no. FR729477.2 for the chromosome and FR745874 for the plasmid) were performed by CLC Genomics Workbench version 6.0.2 (CLC bio, Aarhus, Denmark). On average, 94.8% of the reads were mapped to the bacterial chromosome, while 1.5% and 2.1% of the reads from strains YE-P1 and YE-149, respectively, were mapped against the plasmid. No mapping against the plasmid was performed in strains YE-P4 and YE-150, since they probably lost the pYV plasmid after subculturing. The average coverage ranged between 61.5× and 159.3×. We obtained 86 to 100 contigs (>200 bp in length) for each genome, with a total draft genome size of 4,464,171 to 4,550,830 bp and a G+C content of 47%. Between 4,245 and 4,330 coding sequences (CDSs) and 62 to 64 tRNAs were predicted by genome annotation with Rapid Annotations using Subsystems Technology (RAST) (12).

Hypothetical proteins and prophages are the solely genetic differences identified by preliminary genomic comparison between pig and human *Y. enterocolitica* 4/O:3 isolates. Single nucleotide polymorphism analysis and detailed genome comparison will clarify whether there are different epidemiological origins between clinical and animal isolates.

### Nucleotide sequence accession numbers.

The four whole-genome shotgun projects for the strains YE-P1, YE-P4, YE-149, and YE-150 have been deposited at DDBJ/EMBL/GenBank under the accession no. ASHT01000000, ASHU01000000, ASHV01000000, and ASHW01000000, respectively.
